# Cell-Permeable HSP70 Protects Neurons and Astrocytes Against Cell Death in the Rotenone-Induced and Familial Models of Parkinson’s Disease

**DOI:** 10.1007/s12035-024-04077-9

**Published:** 2024-03-02

**Authors:** Andrey Y. Vinokurov, Alexander A. Palalov, Kristina A. Kritskaya, Svetlana V. Demyanenko, David G. Garbuz, Michael B. Evgen’ev, Noemi Esteras, Andrey Y. Abramov

**Affiliations:** 1 Orel State University, Orel, Russia; 2 Institute of Cell Biophysics of the Russian Academy of Sciences, 142290 Pushchino, Russia; 3 Laboratory of Molecular Neurobiology, Academy of Biology and Biotechnology, Southern Federal University, 344090 Rostov-On-Don, Russia; 4 Engelhardt Institute of Molecular Biology, Russian Academy of Sciences, 119991 Moscow, Russia; 5 Department of Clinical and Movement Neurosciences, UCL Queen Square Institute of Neurology, London, UK; 6 Department of Biochemistry and Molecular Biology, School of Medicine, Complutense University of Madrid, Madrid, Spain

**Keywords:** Hsp70, Mitophagy, Mitochondria, Lysosomes, Parkinson’s Disease

## Abstract

Heat shock protein 70 (HSP70) is activated under stress response. Its involvement in cell protection, including energy metabolism and quality control makes it a promising pharmacological target. A strategy to increase HSP70 levels inside the cells is the application of recombinant HSP70. However, cell permeability and functionality of these exogenously applied proteins inside the cells is still disputable. Here, using fluorescence- labeled HSP70, we have studied permeability and distribution of HSP70 inside primary neurons and astrocytes, and how exogenous HSP70 changes mitochondrial metabolism and mitophagy. We have found that exogenous recombinant HSP70 can penetrate the neurons and astrocytes and distributes in mitochondria, lysosomes and in lesser degree in the endoplasmic reticulum. HSP70 increases mitochondrial membrane potential in control neurons and astrocytes, and in fibroblasts of patients with familial Parkinson´s disease (PD) with PINK1 and LRRK2 mutations. Increased mitochondrial membrane potential was associated with higher mitochondrial ROS production and activation of mitophagy. Importantly, preincubation of the cells with HSP70 protected neurons and astrocytes against cell death in a toxic model of PD induced by rotenone, and in the PINK1 and LRRK2 PD human fibroblasts. Thus, exogenous recombinant HSP70 is cell permeable, and acts as endogenous HSP70 protecting cells in the case of toxic model and familial forms of Parkinson’s Disease.

## Introduction

Heat shock proteins form a group of highly conserved molecules providing the cellular response to stress, and therefore are considered as markers of changes in cells as a result of aging, as well as the development of various pathologies, including neurodegenerative diseases [1–4]. However, an increase in the expression level of these proteins also plays an important role in cell protection [5].

One of the most important groups of heat shock proteins includes several isoform proteins with a molecular weight of 66 to 78 kDa. Some of them are constitutive (HSC70), while the expression of others (HSP70) can be greatly activated under stress [6]. Proteins of this group consist of an N-domain (40 kDa) with nucleotide-binding ability and a C-domain (25 kDa) which binds different substrates. Correct function of HSP70 needs binding to co-chaperones from a family of proteins with a J-domain. One of them, HSP40, is highly expressed in neurons and glial cells [7].

Brain cells and, in particular, neurons with a significant level of metabolism are characterized by a high content of HSC70 and a low level HSP70 [8, 9]. Increase of HSP70 was shown as a result of peripheral nerve cells damage after the axotomy of the facial nerve [6], brain stem death under mevinphos influence [10], high expression of beta-amyloid in primary neuronal cultures [11], and in a model of focal cerebral ischemia [12].

Different methods to induce HSP70 gene expression are known as a way for increasing cell survival after stress conditions. Thus, heat exposure resulted in an increase in HSP70 and a decrease in serotonergic neuronal damage due to the toxic effect of 3,4-methylenedioxymethamphetamine [13].

The protective effect of HSPs can be localized in the cells where they are synthesized as well as directed to surrounding cells and tissues [14]. The ability of HSP70 to cross the blood–brain barrier as it is, or as a part of special constructs [15–18], makes it possible to use exogenous HSP (eHSP) and, in particular, HSP70, for neuroprotection [19].

However, to date, the aspects of translocation, intracellular localization, and the mechanism of eHSP70 influence on the CNS cell metabolism remain a subject of intense research. The penetration ability of eHSP70 depends on the way of administration. It was shown [20] that intraperitoneally injected recombinant human HSP70 was detected in skeletal muscle but not in the brain or spinal cord. But the use of fluorescent or radioactive labeled eHSP70 made it possible to demonstrate the localization of intranasally injected eHSP70 in the hippocampus, cortex, cerebellum and deep brain structures [18, 21, 22].

Here, we have studied trafficking and localization of exogenous fluorescent labeled HSP70 in primary cortical neurons and astrocytes and found a time-dependent distribution of this protein in cellular organelles. HSP70 activated mitochondrial metabolism and mitophagy that protected rotenone-treated neurons and Parkinson’s disease PINK1 and LRRK2 fibroblasts from cell death.

## Materials and Methods

### Cell Cultures

Primary co-culture of cortical neurons and glial cells were prepared from pups 1–3 days postpartum as described [23]. After decapitation, the cortex was removed into ice-cold Versene solution (Gibco, UK), the tissue was minced and trypsinized (Gibco, Canada) (0.25% for 15 min at 37 °C) and plated on polyethylenimine-coated coverslips, and cultured in Neurobasal A medium (Gibco, USA) supplemented with B-27 (Gibco, USA) and 2 mM GlutaMax Supplement (Gibco, USA). Cultures were maintained at 37 °C in a humidified atmosphere of 5% CO_2_ and 95% air, fed twice a week, and maintained for a minimum of 12 days before experimental use.

Neurons were easily distinguishable from glia: they appeared phase bright, had smooth rounded somata and distinct processes, and laid just above the focal plane of the glial layer.

Control human skin fibroblasts and patient’s fibroblasts with the familial Parkinson’s disease-associated mutations PINK1(see more information in [24]) and LRRK2 (see more information in [25]) were used as a research object. The number of passages did not exceed p20.

DMEM (Biological Industries, Kibbutz Beit-Haemek, Israel), 10% FBS (Biological Industries, Kibbutz Beit-Haemek, Israel) with 1% GlutaMAX (Gibco, New York, USA) were applied for cultivation of skin fibroblasts. Cell cultures were maintained at 37 °C in a humidified atmosphere of 5% CO2 and 95% air. The confluence of cells during the studies was 40–50%.

### Exogenous Recombinant HSP70

Recombinant human HSP70 was obtained and purified in the Engelhardt Institute of Molecular Biology of Russian Academy of Sciences. Recombinant human HSP70 included five substitutions in potential glycosylation sites (N35D, S153A, S362A, T419A and T489A), that were created experimentally to reduce protein aggregation and facilitate protein purification from milk of transgenic animals [26]. The coding sequence of HSP70 was cloned into a plasmid obtained from PET-14b to add an N-terminal polyhistidine label (MGSSHHHHHHSSGLVPRGSH). Recombinant HSP70 was expressed in *E. coli* strain BL21. The cells were transfected with a plasmid encoding mutant HSP70, and one colony of the transformed strain was transferred to an LB medium with 150 mcg/ml of ampicillin. Protein expression was induced by the addition of IPTG; after growth for 3 h, cells were collected by centrifugation at 3000 × g for 5 min, the precipitate was stored at -70 °C. The protein was purified in the native state on ice. The cells were treated with ultrasound in 20 ml of buffer A (20 mM NaH2PO4, 500 mM NaCl, 25 mM imidazole, 0.01% NP-40, pH 7.4) with lysozyme (1 mg/ml). The recombinant protein was dialyzed at 4 °C against 1000 times the volume of PBS with 1 mM EDTA to remove traces of Ni^2+^, and then dialyzed again against 10,000 times the volume of PBS. After dialysis, the resulting protein was purified from LPS on polymyxin-agarose (Sigma-Aldrich, USA).

### Fluorescence Labeling of Recombinant Hsp70

In order to estimate the ability of exogenous Hsp70 to enter neurons and to study its intracellular distribution, recombinant Hsp70 was labeled with the Cy5™ monofunctional reactive dye (GE Healthcare). For this purpose, we incubated 1 mg of recombinant Hsp70 with Cy5 in PBS for 30 min in the dark at room temperature. Unbound dye was removed via centrifugation of SigmaSpin columns (S0185-8EA) loaded with 200 μL of labeled protein samples for 4 min at 4000 rpm.

#### Live Cell Imaging

Fluorescence measurements were performed using a Zeiss LSM 900 (Carl Zeiss Microscopy GmbH, Jena, Germany) and a 20 × or 63 × oil immersion objective. Illumination intensity was kept to a minimum (0.1–0.2% of laser output) to avoid phototoxicity.

#### Assessment of the Intracellular Distribution of HSP70

Exogenous recombinant HSP70 labeled by Cy5™ dye (Amersham) was introduced to primary co-cultures of neurons and astrocytes. To determine the permeabilization of HSP70 through the living cell membrane, the primary co-culture was loaded with 5 µM Fluo-4 AM (Invitrogen by Thermo Fisher Scientific, USA). Intracellular distribution of HSP70 was evaluated after labeling mitochondria, lysosomes, and endoplasmic reticulum with the fluorescent probes MitoTracker Green FM (200 nM), LysoTracker Green DND-26 (50 nM) and ER-Tracker Green (1 μM) respectively (all Invitrogen by Thermo Fisher Scientific, USA). Colocalization coefficients for HSP70 and the respective probes were measured using Zen Blue software (Zeiss), which indicates the relative number of colocalized pixels in channel 1 in relation to the total number of pixels above the threshold value.

#### Measurement of the Mitochondrial Membrane Potential

The mitochondrial membrane potential (ΔΨm) was measured by loading of cells with 25 nM tetramethylrhodamine methyl ester (TMRM) (Invitrogen by Thermo Fisher Scientific, USA) in a HEPES-buffered HBSS for 45 min at a room temperature. Z-stack images were obtained by confocal microscopy, and the maximum fluorescence intensity was used as a measure of ΔΨm.

*Mitochondrial ROS production* was measured with 1 μM MitoTracker Red CM-H_2_-XRos (Invitrogen, Oregon, USA) using a previously described protocol [27]. Cells were loaded with the probe for 10 min which was kept in the solution during the experiments to avoid the intracellular accumulation of the oxidized probe.

#### Measurement of Mitophagy

Mitophagy level was calculated as the degree of colocalization of mitochondria and lysosomes [28]. Cells were loaded with 200 nM MitoTracker Green FM and 50 nM LysoTracker Red DND-99 in HBSS for 30 min before the experiments. Colocalization coefficients for mitochondria and lysosomes were measured using Zen Blue software (Zeiss), which indicates the relative number of colocalized pixels in channel 1 in relation to the total number of pixels above the threshold value.

#### Cell Death. Necrosis

The measurement of necrotic cells death was carried out using Hoechst 33,342 and Propidium Iodide. Cells were incubated with 5 μM Hoechst 33,342 (Invitrogen, Oregon, USA) and 20 μM Propidium iodide (PI) (Invitrogen, Oregon, USA) for 30 min at 37℃. While Hoechst is a blue fluorescent dye that stains chromatin DNA, PI is only permeable to dead cells and shows red fluorescence, so it is possible to calculate the percentage of dead cells (showing red fluorescence) versus total number of cells (showing blue fluorescence). *Apoptosis* was detected among dead cells based on morphological features: the presence of a fragmented nucleus, karyopyknosis, karyorexis, apoptotic bodies.

## Apoptosis

NucView™ 488 Caspase-3 Substrate (Biotium) (excitation/emission maxima ~ 488/515 nm) was applied for detection of caspase-3/7 activity and visualization of morphological changes in the nucleus during apoptosis. Cells were incubated with 5 µM NucView™ 488 Caspase-3 Substrate 30 min at room temperature (Bryanskaya et al., 2024).

## Statistical Analysis

OriginPro 2019 (Microcal Software Inc., Northampton, MA) was used for graphs generation and statistical analysis. The Mann–Whitney U test was used to estimate the statistical significance between experimental groups, if not stated otherwise. One-way ANOVA was used for experiments of dead cells. Sample sizes for experiments were selected to capture adequate technical variation (number of cells; number of fields of view; number of coverslips). Significance was accepted at a 95% confidence level (p < 0.05). Data are given as mean ± SEM.

## Results

### Exogenous Recombinant Human HSP70 Can Penetrate the Cell Membrane

**Fig. 1:**
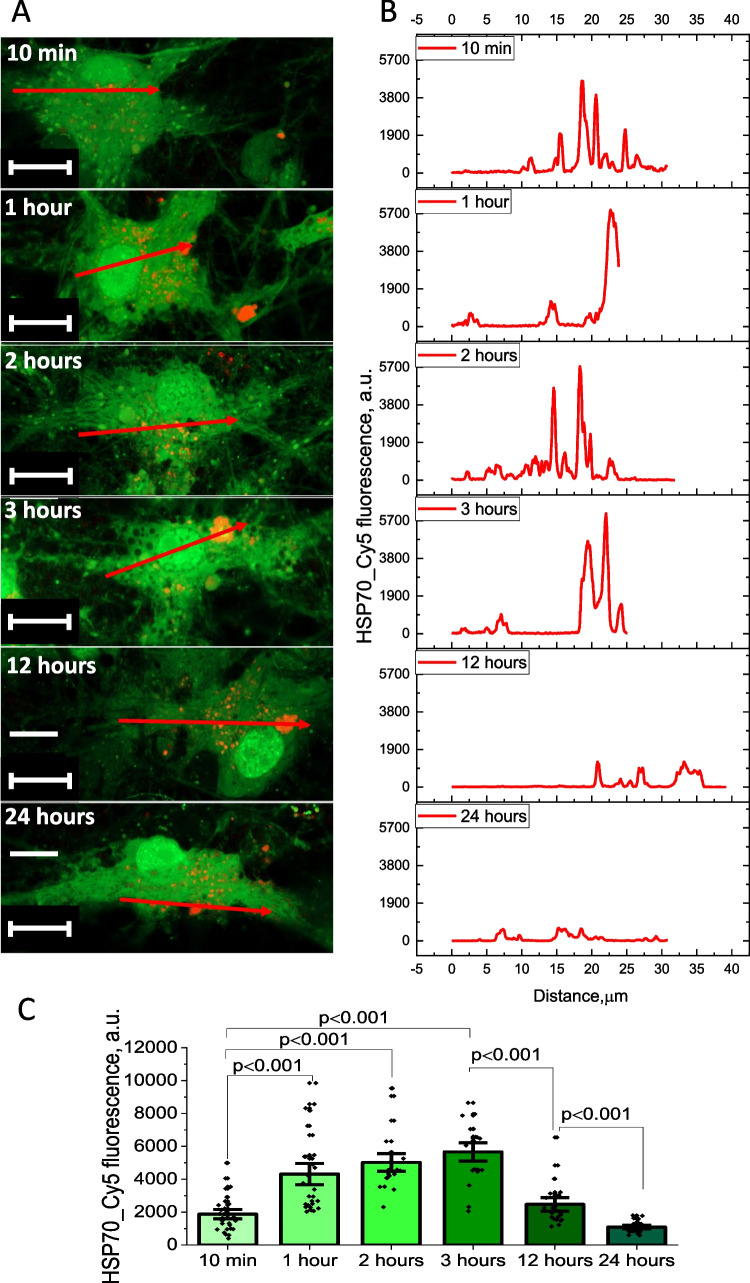
Exogenous HSP70 accumulates in live brain cells during the first 24 h of incubation. Time-dependent penetration of HSP70 conjugated with Cy5 into the cells of primary neuroglial co-culture (shown astrocytes), labeled with Fluo-4 AM (**A**) and corresponding fluorescence intensity profiles (**B**) in different time after HSP70-Cy5 application. **C**- Changes in HSP70-Cy5 fluorescence in neurons and astrocytes during 24 h after applications taken as a maximal values of fluorescence in Z-stack images for the red channel. Scalebar 10 μm. Data presented as mean ± SEM

Short incubation of the primary neurons and astrocytes with exogenous recombinant HSP70 labeled with Cy5 led to the penetration of this peptide through the cell membrane inside the primary cortical neurons and astrocytes. However, HSP70 was not equally distributed inside the cells with peaks of intensity of fluorescence which was hundred times higher than in cytosol (Fig. 1A-B). Fluorescence intensity in neurons and astrocytes progressively increases after 2 and 3 h of incubation (Fig. 1A, C). However, after 12 h incubation with HSP70, Cy5 fluorescence was lower, and decreased to a minimum after 24 h (Fig. 1C). It should be noted that we have found no difference in HSP70 penetration between neurons and astrocytes. Thus, recombinant human HSP70 is able to penetrate the cells with a maximal content after 3 h of incubation.

### Exogenous HSP70 Distributes Unevenly Inside Different Cellular Compartments of Primary Neurons and Astrocytes

**Fig. 2:**
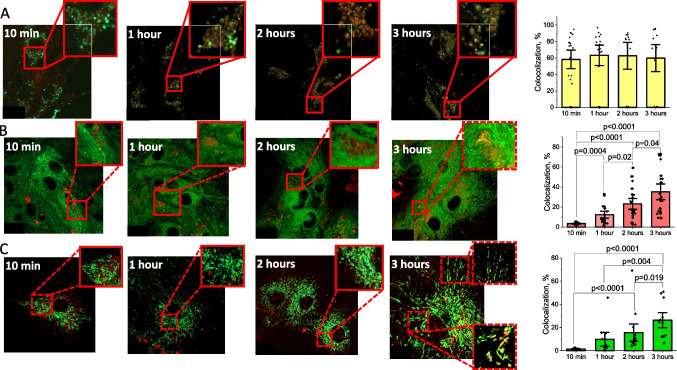
Exogenous HSP70 distributes inside intracellular compartments in primary neurons and astrocytes. HSP70 conjugated with Cy5 strongly colocalizes with lysosomes (**A**). A lower but statistically significant degree of colocalization was detected for HSP70 with endoplasmic reticulum (**B**), and mitochondria (**C**) using Z-stack images with red and green channels. Ai, Bi, Ci are summaries colocalization coefficients (which indicates the relative number of colocalized pixels in channel 1 in relation to the total number of pixels above the threshold value) of HSP70-Cy5 with lysosomes, endoplasmic reticulum and mitochondria of cortical neurons and astrocytes. Scalebar 10 μm. Data presented as mean ± SEM

In order to identify the subcellular localization of the high content of exogenous HSP70 internalized by the cells, we have studied the co-localization of HSP70-Cy5 with three major cellular organelles: lysosomes, mitochondria, and endoplasmic reticulum in primary co-cultures of cortical neurons and astrocytes. Application of the recombinant fluorescent labeled peptide led to the colocalization of HSP70-Cy5 and LysoTracker Green DND-26 (Fig. 2A). The percentage of colocalization did not significantly change after 1, 2, and 3 h of incubation. Thus, colocalization coefficient for HSP70 and lysosomes was 58.3 ± 7.5%, N = 3 experiments; n = 19 neurons and astrocytes (10 min incubation), 63.3 ± 8.2%, N = 3 experiments, n = 16 cells (1 h incubation), 62.7 ± 10.9%, N = 3 experiments, n = 12 cells (2 h of incubation), 59.9 ± 10.9%, N = 3 experiments, n = 12 cells (3 h of incubation; Fig. 2A).

The accumulation of HSP70-Cy5 in the endoplasmic reticulum (ER-Tracker Green) was lower compared to lysosomes and was dependent on the time of incubation. Thus, colocalization coefficient for 1 h of incubation was 12.3 ± 2.3%, N = 3 experiments, n = 17 cells, for 2 h increased to 23.1 ± 3.6%, N = 3 experiments, n = 20 cells, and for 3 h of incubation rose to 35.2 ± 5.2%, N = 3 experiments, n = 19 cells (Fig. 2B).

Incubation of primary neurons and astrocytes with HSP70-Cy5 also induced colocalization with mitochondria (MitoTracker Green), and its accumulation in this organelle was also time-dependent, reaching its maximum after 3 h of incubation (colocalization coefficient 9.9 ± 4.0%, N = 3 experiments, n = 11 cells, 15.6 ± 4.9%, N = 3 experiments, n = 13 cells, 26.4 ± 4.3%, N = 3 experiments, n = 14 cells from 1 to 3 h, respectively (Fig. 2C).

### Exogenous HSP70 Increases ΔΨm

**Fig. 3:**
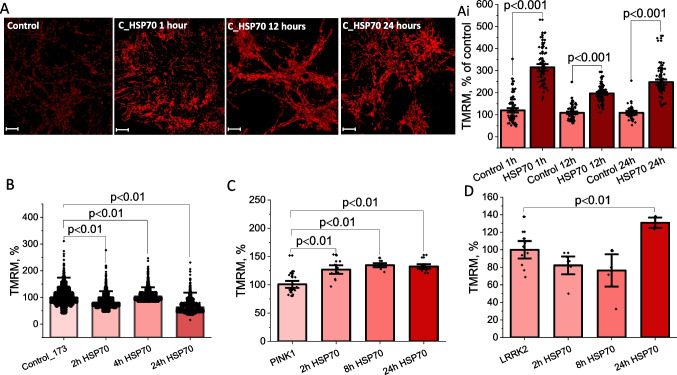
HSP70 changes ΔΨm in primary neurons and astrocytes and Parkinson’s disease fibroblasts. Effect of C-terminal fragment of HSP70 (C_HSP70) on mitochondrial membrane potential (ΔΨm -TMRM fluorescence intensity taken as a maximal values of fluorescence in Z-stack images) in primary cortical co-cultures of neurons and astrocytes (A and Ai). Effect of C_HSP70 on TMRM intensity in control fibroblasts (B, control taken as 100%) and fibroblasts with PINK1 (C, untreated PINK1 taken as 100%) and LRKK2 (D, untreated LRRK2 taken as 100%) mutations associated with familial Parkinson’s disease. Scalebar 10 μm. Data presented as mean ± SEM. Significance was accepted at a 95% confidence level (p < 0.05)

Mitochondrial membrane potential (ΔΨm) is vitally important to sustain its function, and considering this, Δψm can be used as an indicator of mitochondrial health. We used TMRM as a fluorescent indicator for ΔΨm in primary co-culture neurons and astrocytes and in fibroblasts, as previously described [29]. Primary cortical co-cultures were incubated with the C-terminal fragment of HSP70 (C_HSP70) obtained with a construction based on the pET28 vector encoding a fragment of HSP70 (385–641 amino acid residues). 1 h of incubation cells with C_HSP70 induced a significant increase in ΔΨm compared to untreated control cells in the primary cortical co-culture, and similar data were obtained after 12 and 24 h of incubation (N = 3 experiments, n = 40–60 cells for each group (Fig. 3A). Treatment of control fibroblasts with full HSP70 induced a mirror decrease in ΔΨm in the in first 2 h of incubation followed by recovery of Δψm after 4 h of incubation with this peptide (N = 5 experiments; Fig. 3B). In contrast, fibroblasts with Parkinson’s mutations in PINK1 and LRRK2 demonstrated a significant increase in ΔΨm after 24 h of incubation (N = 5 experiments for PINK1, N = 4 experiments for LRRK2) (Fig. 3C). Thus, exogenous HSP70 could activate mitochondrial membrane potential which results in an increase of Δψm.

### HSP70 Modifies Mitochondrial ROS Production

**Fig. 4:**
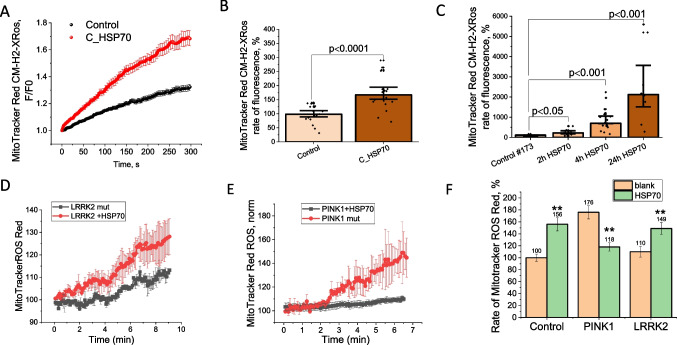
HSP70 increases mitochondrial ROS production in primary neurons, astrocytes and Parkinson’s disease fibroblasts. Average traces of MitoTracker Red CM-XRos fluorescence, which indicates mitochondrial ROS production in control cells and cells after 1 h incubation with C_HSP70 in primary cortical co-culture (**A**). **B**- The rates of mitochondrial ROS production in primary neurons and astrocytes (normalized to control). **C**- the rate of the mitochondrial ROS production in control fibroblasts depending on the time of incubation with HSP70 (normalized to untreated control). Average traces of MitoTracker Red CM-H2 X ROS fluorescence in fibroblasts with LRRK2 mutation (**D**) or PINK1 mutation (**E**) with and without 24 h incubation with HSP70. The rate of mitochondrial ROS production in control, PINK1 mutation and LRRK2 mutation fibroblasts (untreated control was taken as 100%) treated and untreated with HSP70 for 24 h. Data presented as mean ± SEM. **p < 0.01

Mitochondrial ROS production in neurons and astrocytes depends on a number of metabolic parameters including ΔΨm [30]. Incubation of the primary neuroglial co-culture with C_HSP70 (1 h) increased the mitochondrial ROS production measured with the fluorescent indicator MitoTracker Red CM-H_2_-XRos (N = 3 experiments; Fig. 4A, B).

It should be noted that application of the full HSP70 to control fibroblasts induced an increase in the rate of mitochondrial ROS production in the same way as C_HSP70 in a co-culture of neurons and astrocytes (N = 5 experiments; Fig. 4C). Prolonged incubation (up to 24 h) of the full HSP70 with fibroblasts with LRRK2 mutation also induced elevation of the mitochondrial ROS production (N = 4 experiments; Fig. 4D, F). However, in contrast to control and LRRK2 mutation, incubation of the fibroblast with PINK1 mutation with exogenous HSP70 induced decrease in mitochondrial ROS production (Fig. 4E-F). Thus, both—C_HSP70 and full HSP70 activate mitochondrial ROS production in fibroblasts and in co-culture of neurons and astrocytes.

### Exogenous HSP70 Induces Mitophagy in Neurons and Astrocytes and Fibroblasts with PINK1 Mutation

**Fig. 5:**
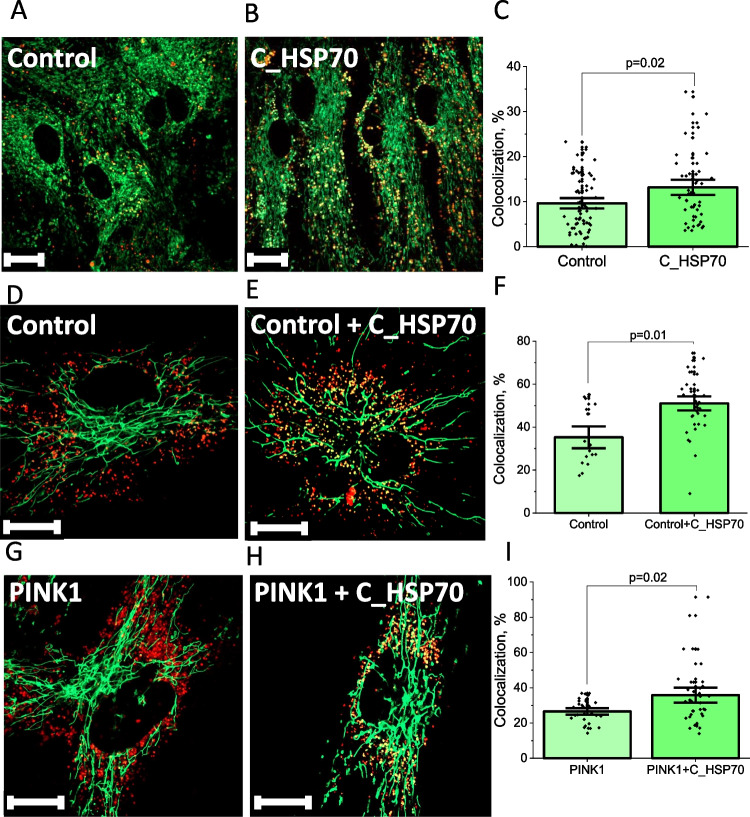
Exogenous HSP70 induces mitophagy in neurons, astrocytes and fibroblasts. Colocalization of MitoTracker Green and LysoTracker Red in control primary neuroglial culture (**A**) and after 24 h of incubation with C_HSP70 (**B**), percentage of colocalization in **C**. Colocalization of MitoTracker Green and LysoTracker Red in control fibroblasts (**D**) and after 24 h of incubation with C_HSP70 (**E**), increase percentage of colocalization (**F**). Colocalization of MitoTracker Green and LysoTracker Red in PINK1-mutated fibroblasts (**G**) and after 24 h of incubation with C_HSP70 (H), increase percentage of colocalization (**J**) scalebar 10 μm. Data presented as mean ± SEM. Significance was accepted at a 95% confidence level (p < 0.05)

Mitophagy is a cellular physiological process in which mitochondria are selectively degraded by autophagy. It is essential for mitochondrial renewal and prevention of different pathological conditions, such as hypoxia and oxidative damage. After 24 h of incubation with C_HSP70, we detected a significantly higher mitophagy level in the primary co-culture of neurons and astrocytes, as determined by colocalization of MitoTracker Green FM and LysoTracker Red DND-99 as described (Fig. 5A-C). The colocalization coefficients of mitochondria and lysosomes were 9.6 ± 0.8% and 13.2 ± 1.1% for control cells and after 24 h of incubation with C_HSP70, respectively (N = 3 experiments, n = 69 cells after C_HSP70 incubation, n = 50 control cells).

It should be noted that C_HSP70 induced mitophagy not only in neurons and astrocytes but also in fibroblasts. Thus, 24 h of incubation of control fibroblasts with C_HSP70 increased colocolisation of mitochondria and lysosomes from 34 ± 4% to 56 ± 7%, N = 5 experiments; Fig. 5D, E, F). Importantly, C_HSP70 induced mitophagy even in fibroblasts of patients with PINK1 mutation (Fig. 5G, H, J).

### Exogenous HSP70 Protects Neurons and Glia Against Rotenone-Induced Neurotoxicity

**Fig. 6:**
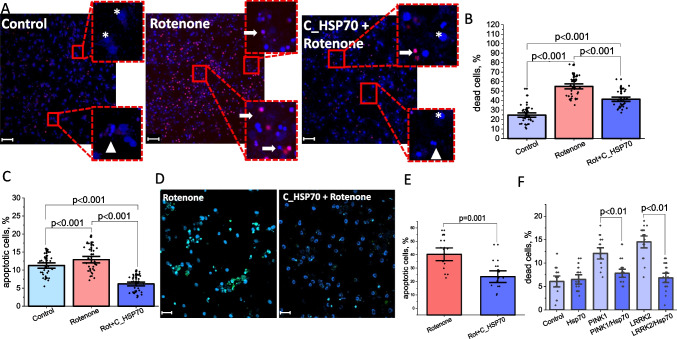
Exogenous HSP70 indices cell protection against rotenone-induced neurotoxicity and in familial Parkinson’s’ disease fibroblast models. Primary neuroglial co-culture labeled with Hoechst 33,342 (blue, labelling all nuclei) and PI (red, labelling nuclei of necrotic cells): control culture, after 5 μM rotenone incubation and after rotenone and C_HSP70 24 h incubation (**A**). The arrows indicates fragmented nuclei. Percentage of necrotic cells in the primary neuroglial co-culture (**B**), cells with apoptotic features in the primary neuroglial co-culture (**C**). Images of the Hoechst 33,342 (blue, labelling all nuclei) and NucView488 (green) of primary co-cultures of neurons and astrocytes treated with 5 μM rotenone for 6 h and with preincubation with HCP70. **D**- percentage of NucView 488 positive neurons and astrocytes. The number of dead cells (PI positive necrotic cells) in control, PINK1-, and LRRK2-mutated fibroblasts before and after incubation with HSP70 (**F**). One-way ANOVA was used for statistics. Scalebar 100 μm. Significance was accepted at a 95% confidence level (p < 0.05)

Rotenone, a nonselective inhibitor of the mitochondrial complex I, is widely used to induce a toxic cellular model of Parkinson's disease [31]. We therefore, employed this method to mimic PD in our cells and test the protective capacity of HSP70 against necrotic and apoptotic cell death in the pathology. 24 h incubation of the primary cortical neuroglial co-culture with 5 μM rotenone increased (up to 55 ± 1.8%) the number of PI-labeled cells, indicating necrotic cells (Fig. 6A-B). Pre-incubation of the cells with C_HSP70 significantly decreased the percentage of necrotic cells, although it did not reach the level of the necrotic cells in the control culture (24.7 ± 1.5%, in the rotenone-induced neurotoxicity model and 41.4 ± 8% of cells incubated with rotenone and C_HSP70, n = 3 experiments for each group; p < 0.0001; Fig. 6A, B).

Apoptosis was detected based on the morphological features described previously [32]. In control cultures 11.3 ± 0.5% of the dead cells had fragmented nuclei, suggesting apoptosis in these cells. In the rotenone-induced neurotoxicity model, the percentage of apoptotic cells was significantly higher – 12.9 ± 0.6%. Pre-incubation with C_HSP70, reduced the number of cells undergoing apoptosis to 6.1 ± 0.4%, which was significantly lower in comparison with the rotenone group and the control culture (N = 3 experiments, p < 0.0001) (Fig. 6C). In order to prove the protective effect of exogenous HSP70 against rotenone-induced apoptosis we used fluorescent substrate for caspase-3, NucView 488, which becomes fluorescent and binds to nuclear DNA upon activation of the key apoptotic enzyme caspase-3 (Bryanskaya et al. 2023). Incubation of the primary neurons and astrocytes with 5 μM rotenone for 6 h induced appearance of the NucView 488 signal in the nuclei of 40.4 ± 5% of cells (Fig. 6D, E). Pre-incubation of the neurons and astrocytes with HSP70 significantly reduced percentage of NucView 488 positive cells (to 21.6 ± 1.4%, N = 3 experiments, p < 0.001; Fig. 6D, E) that confirmed previously shown anti-apoptotic effect of HSP70 in glial cells (DOI: 10.3390/cells11010093).

### Exogenous HSP70 Protects Fibroblasts with PINK1 or LRRK2 Mutations

In the case of the human fibroblasts, incubation of the cells with full peptide HSP70 did not change the percentage of PI-labeled cells in control fibroblasts (6.1 ± 0.8% versus 6.5 ± 0.7% after 24 h of HSP70 incubation, N = 14 experiments, Fig. 6F). However, HSP70 significantly decreased the percentage of necrotic cells in the fibroblasts with PINK1 mutation (from 12.1 ± 0.8% to 7.8 ± 0.6% after 24 h of HSP70 incubation, N = 3 experiments) and LRRK2 mutations (14.5 ± 0.9% versus 6.8 ± 0.7% after 24 h of HSP70 incubation) (p < 0.01) Fig. 6F), indicating that HSP70 protects against cell death also in the familial model of PD.

## Discussion

Here we have shown that recombinant HSP70 is able to pass plasma membrane and distribute in the cytosol and cellular organelles. It should be noted that the concentration of HSP70 is not stable and starts to reduce after 3 h of incubation. Considering high colocalization of the fluorescent HSP70 with lysosomes, we can suggest it induces the activation of the autophagic removal of unwanted exogenous proteins including recHSP70 [33]. However, physiological activity of HSP70 after 3 h, with maximal values in 24 h of incubation, when the level of HSP70 is relatively low, suggests that for activation of HSP70-dependent processes the concentration of this peptide should not be very high. Previous studies suggested that exogenous HSP70 diffusion into the cells depends on the cell type [21] and the most available data indicates the perinuclear zone localization of eHSP70 in brain cells under stress conditions [21, 22], similar to the localization of the endogenous HSP70 [34].

It should be noted that we have not found any differences in the action of full peptide and of C-terminal fragment of HSP70 (C_HSP70) obtained with a construction based on the pET28 vector encoding a fragment of HSP70 (385–641 amino acid residues). Both peptides increased mitochondrial membrane potential, activated mitochondrial ROS production in neurons and fibroblasts and demonstrated the ability to protect cell against toxic (rotenone) or familial forms of Parkison’s disease.

Although intracellular localization of the endogenous HSP70 is disputable on the basis of the functional activity, we suggest that exogenous HSP70 is mostly localized in the same organelles as endogenous HSP70 and exercises similar functions as lysosomal membrane stabilizer, activator of mitophagy [35] and as a protector against ER stress [36].

Exogenous HSP70 is able to increase ΔΨm in neurons, astrocytes and fibroblasts in a similar way as the endogenous protein improves energy metabolism [37]. It should be noted, that the more pronounced effect of HSP70 on ΔΨm with fibroblasts with PD could be explained by the restoration of the potential in these cells, since basal mitochondrial membrane potential in PINK1 and LRRK2 mutated fibroblasts is lower compared to control cells [25, 38, 39].

Mitochondrial ROS production depends on mitochondrial membrane potential and very often increases with hyperpolarization, which plays a signaling role and may be a trigger of pathology [30, 40]. Considering this, the increase of ΔΨm induced by HSP70 in primary cortical co-cultures and in fibroblasts could be a trigger for mitochondrial ROS production. The decrease in the rate of mitochondrial ROS production observed in fibroblasts with the PINK1 mutation could be due to the restoration of normal mitochondrial function in these cells [41].

Protection of the cells from toxic (rotenone) or familial forms of Parkinsons’ disease models could be due to activation of autophagy, mitophagy and mitochondrial metabolism in these cells. It t is not surprising that this cytoprotection was higher in the cells with pathology, where all these processes are corrupted.

## Data Availability

Data will be made available under the reasonable request.

## References

[1] Hu D, Chen F, Guan C, Yang F, Qu Y (2013) Anti-hypoxia effect of adenovirus-mediated expression of heat shock protein 70 (HSP70) on primary cultured neurons. J Neurosci Res 91(9):1174–1182. 10.1002/jnr.23240 23686726 10.1002/jnr.23240

[2] Bobkova NV et al (2015) Exogenous Hsp70 delays senescence and improves cognitive function in aging mice. Proc Natl Acad Sci U S A 112(52):16006–16011. 10.1073/pnas.1516131112 26668376 10.1073/pnas.1516131112 PMC4702952

[3] Bechtold DA, Rush SJ, Brown IR (2000) Localization of the heat-shock protein Hsp70 to the synapse following hyperthermic stress in the brain. J Neurochem 74(2):641–646. 10.1046/j.1471-4159.2000.740641.x 10646515 10.1046/j.1471-4159.2000.740641.x

[4] Kim HJ, Jung JI, Kim Y, Lee JS, Yoon YW, Kim J (2010) Loss of hsp70.1 decreases functional motor recovery after spinal cord injury in mice. Korean J Physiol Pharmacol 14(3):157–161. 10.4196/kjpp.2010.14.3.157 20631888 10.4196/kjpp.2010.14.3.157 PMC2902807

[5] Kim HP, Morse D, Choi AMK (2006) Heat-shock proteins: new keys to the development of cytoprotective therapies. Expert Opin Ther Targets 10(5):759–769. 10.1517/14728222.10.5.759 16981832 10.1517/14728222.10.5.759

[6] Moreno-Flores MT, Olazábal UE, Kreutzberg GW (1997) Axotomy increases the expression of glucose-regulated protein 78 kDa in rat facial nucleus. Exp Neurol 146(1):10–16. 10.1006/exnr.1997.6526 9225733 10.1006/exnr.1997.6526

[7] Geraci F, Turturici G, Sconzo G (2011) Hsp70 and its molecular role in nervous system diseases. Biochem Res Int 2011. 10.1155/2011/618127 10.1155/2011/618127 PMC3049350 21403864

[8] Tidwell JL, Houenou LJ, Tytell M (2004) Administration of Hsp70 in vivo inhibits motor and sensory neuron degeneration. Cell Stress Chaperones 9(1):88–98. 10.1379/1466-1268(2004)009%3c0088:AOHIVI%3e2.0.CO;2 15270081 10.1379/CSC-9R.1 PMC1065310

[9] Moon IS, Park IS, Schenker LT, Kennedy MB, Il Moon J, Jin I (2001) Presence of both constitutive and inducible forms of heat shock protein 70 in the cerebral cortex and hippocampal synapses. Cereb Cortex 11(3):238–248. 10.1093/cercor/11.3.238 10.1093/cercor/11.3.238 11230095

[10] Chan JYH et al (2007) Heat shock protein 60 or 70 activates Nitric-Oxide Synthase (NOS) I- and inhibits NOS II-associated signaling and depresses the mitochondrial apoptotic cascade during brain stem death. J Biol Chem 282(7):4585–4600. 10.1074/jbc.M603394200 17150954 10.1074/jbc.M603394200

[11] Magrané J, Smith RC, Walsh K, Querfurth HW (2004) Heat shock protein 70 Participates in the neuroprotective response to intracellularly expressed β-amyloid in neurons. J Neurosci 24(7):1700–1706. 10.1523/JNEUROSCI.4330-03.2004 14973234 10.1523/JNEUROSCI.4330-03.2004 PMC6730449

[12] da C. Tirapelli DP, Carlotti Junior CG, Leite JP, Tirapelli LF, Colli BO (2010) Expression of HSP70 in cerebral ischemia and neuroprotetive action of hypothermia and ketoprofen. Arq Neuropsiquiatr 68(4):592–596. 10.1590/S0004-282X2010000400021 10.1590/s0004-282x2010000400021 20730315

[13] Escobedo I, Peraile I, Orio L, Colado MI, O’Shea E (2007) Evidence for a role of Hsp70 in the neuroprotection induced by heat shock pre-treatment against 3,4-methylenedioxymethamphetamine toxicity in rat brain. J Neurochem 101(5):1272–1283. 10.1111/j.1471-4159.2007.04459.x 17328712 10.1111/j.1471-4159.2007.04459.x

[14] Lasek RJ, Gainer H, Barker JL (1977) Cell-to-cell transfer of glial proteins to the squid giant axon. The glia-neuron protein transfer hypothesis. J Cell Biol 74(2):501–523. 10.1083/jcb.74.2.501 885913 10.1083/jcb.74.2.501 PMC2110074

[15] Kwon HJ et al (2019) Heat shock protein 70 increases cell proliferation, neuroblast differentiation, and the phosphorylation of CREB in the hippocampus. Lab Anim Res 35(1):1–11. 10.1186/s42826-019-0020-2 32257909 10.1186/s42826-019-0020-2 PMC7081702

[16] Doeppner TR, Kaltwasser B, Fengyan J, Hermann DM, Bähr M (2013) TAT-Hsp70 induces neuroprotection against stroke via anti-inflammatory actions providing appropriate cellular microenvironment for transplantation of neural precursor cells. J Cereb Blood Flow Metab 33(11):1778–1788. 10.1038/jcbfm.2013.126 23881248 10.1038/jcbfm.2013.126 PMC3824176

[17] Tunesi M et al (2019) Hydrogel-based delivery of Tat-fused protein Hsp70 protects dopaminergic cells in vitro and in a mouse model of Parkinson’s disease. NPG Asia Mater 11(1). 10.1038/s41427-019-0128-8

[18] Shevtsov MA et al (2014) Neurotherapeutic activity of the recombinant heat shock protein Hsp70 in a model of focal cerebral ischemia in rats. Drug Des Dev Ther 8:639–650. 10.2147/DDDT.S62024 10.2147/DDDT.S62024 PMC4044995 24920887

[19] Ekimova IV, Nitsinskaya LE, Romanova IV, Pastukhov YF, Margulis BA, Guzhova IV (2010) Exogenous protein Hsp70/Hsc70 can penetrate into brain structures and attenuate the severity of chemically-induced seizures. J Neurochem 115(4):1035–1044. 10.1111/j.1471-4159.2010.06989.x 20831598 10.1111/j.1471-4159.2010.06989.x

[20] Gifondorwa DJ et al (2007) Exogenous delivery of heat shock protein 70 increases lifespan in a mouse model of amyotrophic lateral sclerosis. J Neurosci 27(48):13173–13180. 10.1523/JNEUROSCI.4057-07.2007 18045911 10.1523/JNEUROSCI.4057-07.2007 PMC6673412

[21] Yurinskaya M et al (2015) The fate of exogenous human HSP70 introduced into animal cells by different means. Curr Drug Deliv 12(5):524–532. 10.2174/1567201812666150724094207 26205901 10.2174/1567201812666150724094207

[22] Bobkova NV et al (2014) Therapeutic effect of exogenous Hsp70 in mouse models of Alzheimer’s disease. J Alzheimer’s Dis 38(2):425–435. 10.3233/JAD-130779 23985416 10.3233/JAD-130779

[23] Esteras N, Kundel F, Amodeo GF, Pavlov EV, Klenerman D, Abramov AY (2021) Insoluble tau aggregates induce neuronal death through modification of membrane ion conductance, activation of voltage-gated calcium channels and NADPH oxidase. FEBS J 288(1):127–141. 10.1111/febs.15340 32338825 10.1111/febs.15340

[24] Komilova NR et al (2022) Metabolically induced intracellular pH changes activate mitophagy, autophagy, and cell protection in familial forms of Parkinson’s disease. FEBS J 289(3):699–711. 10.1111/febs.16198 34528385 10.1111/febs.16198

[25] Ludtmann MHR, Kostic M, Horne A, Gandhi S, Sekler I, Abramov AY (2019) LRRK2 deficiency induced mitochondrial Ca 2+ efflux inhibition can be rescued by Na + /Ca 2+ /Li + exchanger upregulation. Cell Death Dis 10(4). 10.1038/s41419-019-1469-5 10.1038/s41419-019-1469-5 PMC6424963 30890692

[26] Gurskiy YG et al (2016) The development of modified human Hsp70 (HSPA1A) and its production in the milk of transgenic mice. Cell Stress Chaperones 21(6):1055–1064. 10.1007/s12192-016-0729-x 27511022 10.1007/s12192-016-0729-x PMC5083674

[27] Angelova PR, Dinkova-Kostova AT, Abramov AY (2021) Assessment of ros production in the mitochondria of live cells. Methods Mol Biol 2202:33–42. 10.1007/978-1-0716-0896-8_2 32857343 10.1007/978-1-0716-0896-8_2

[28] Berezhnov AV et al (2016) Intracellular pH modulates autophagy and mitophagy. J Biol Chem 291(16):8701–8708. 10.1074/jbc.M115.691774 26893374 10.1074/jbc.M115.691774 PMC4861439

[29] Vinokurov AY et al (2023) HPRT1 deficiency induces alteration of mitochondrial energy metabolism in the brain. Mol Neurobiol 60(6):3147–3157. 10.1007/s12035-023-03266-2 36802322 10.1007/s12035-023-03266-2 PMC10122629

[30] Angelova PR, Abramov AY (2016) Functional role of mitochondrial reactive oxygen species in physiology. Free Radic Biol Med 100:81–85. 10.1016/j.freeradbiomed.2016.06.005 27296839 10.1016/j.freeradbiomed.2016.06.005

[31] Fitzgerald JC et al (2014) Monoamine oxidase-A knockdown in human neuroblastoma cells reveals protection against mitochondrial toxins. FASEB J 28(1):218–229. 10.1096/fj.13-235481 24051032 10.1096/fj.13-235481

[32] Novikova IN, Potapova EV, Dremin VV, Dunaev AV, Abramov AY (2022) Laser-induced singlet oxygen selectively triggers oscillatory mitochondrial permeability transition and apoptosis in melanoma cell lines. Life Sci 304(April). 10.1016/j.lfs.2022.120720 10.1016/j.lfs.2022.120720 35716733

[33] Patwardhan CA et al (2023) Capsaicin binds the N-terminus of Hsp90, induces lysosomal degradation of Hsp70, and enhances the anti-tumor effects of 17-AAG (Tanespimycin). Sci Rep 13(1):1–15. 10.1038/s41598-023-40933-9 37612326 10.1038/s41598-023-40933-9 PMC10447550

[34] Bodega G, Hernández C, Suárez I, Martín M, Fernández B (2002) HSP70 constitutive expression in rat central nervous system from postnatal development to maturity. J Histochem Cytochem 50(9):1161–1168. 10.1177/002215540205000902 12185193 10.1177/002215540205000902

[35] Zheng Q et al (2018) Hsp70 participates in PINK1-mediated mitophagy by regulating the stability of PINK1. Neurosci Lett 662(October 2017):264–270. 10.1016/j.neulet.2017.10.051 10.1016/j.neulet.2017.10.051 29107085

[36] Xie WY, Zhou XD, Yang J, Chen LX, Ran DH (2016) Inhibition of autophagy enhances heat-induced apoptosis in human non-small cell lung cancer cells through ER stress pathways. Arch Biochem Biophys 607:55–66. 10.1016/j.abb.2016.08.016 27565443 10.1016/j.abb.2016.08.016

[37] Rodriguez YA, Kaur S, Nolte E, Zheng Z, Blagg BSJ, Dobrowsky RT (2021) Novologue therapy requires heat shock protein 70 and thioredoxin-interacting protein to improve mitochondrial bioenergetics and decrease mitophagy in diabetic sensory neurons. ACS Chem Neurosci 12(16):3049–3059. 10.1021/acschemneuro.1c00340 34340312 10.1021/acschemneuro.1c00340 PMC8456717

[38] Gandhi S et al (2009) PINK1-associated Parkinson’s disease is caused by neuronal vulnerability to calcium-induced cell death. Mol Cell 33(5):627–638. 10.1016/j.molcel.2009.02.013 19285945 10.1016/j.molcel.2009.02.013 PMC2724101

[39] Abramov AY, Gegg M, Grunewald A, Wood NW, Klein C, Schapira AHV (2011) Bioenergetic consequences of PINK1 mutations in parkinson disease. PLoS One 6(10). 10.1371/journal.pone.0025622 10.1371/journal.pone.0025622 PMC3197155 22043288

[40] Esteras N, Rohrer JD, Hardy J, Wray S, Abramov AY (2017) Mitochondrial hyperpolarization in iPSC-derived neurons from patients of FTDP-17 with 10+16 MAPT mutation leads to oxidative stress and neurodegeneration. Redox Biol 12(February):410–422. 10.1016/j.redox.2017.03.008 28319892 10.1016/j.redox.2017.03.008 PMC5357682

[41] Gandhi S, Vaarmann A, Yao Z, Duchen MR, Wood NW, Abramov AY (2012) Dopamine Induced Neurodegeneration in a PINK1 Model of Parkinson’s Disease. PLoS One 7(5):e37564–e37564. 10.1371/journal.pone.0037564 22662171 10.1371/journal.pone.0037564 PMC3360782

